# Supplementary material for: Are men’s gender equality beliefs associated with self-reported intimate partner violence perpetration? A state-level analysis of California men
Source: PLoS One. 2025 Jan 15;20(1):e0315293. doi: 10.1371/journal.pone.0315293 (PMC11734971; doi:10.1371/journal.pone.0315293)
Supplement: S1 File — (PDF) [file pone.0315293.s002.pdf]

```
// Are men‚Äôs gender equality beliefs associated with self-reported intimate partner violence perpetration? A state-level analysis of California men
// Final/clean analysis code - can be shared
// Nicole Johns - nejohns@health.ucsd.edu
// 10.22.24

// Data are available for download in .dta format from OpenICPSR
// 2021 CalVEX data: https://www.openicpsr.org/openicpsr/project/204402/version/V1/view
// 2022 CalVEX data: https://www.openicpsr.org/openicpsr/project/204401/version/V1/view
// 2023 CalVEX data: https://www.openicpsr.org/openicpsr/project/199087/version/V1/view

// Setup
    clear all
    set more off

// Combine datasets
    use "CalVEX2021 OpenICPSR dataset v1.dta", clear
    gen year=2021
    append using "CalVEX2022 OpenICPSR dataset v1.dta"
    replace year=2022 if year==.
    append using "CalVEX2023 OpenICPSR dataset v1.dta"
    replace year=2023 if year==.

// Keep only men respondents
    keep if GENDER_2==2
    di _N // 3658

// Weighting
    svyset [pweight=WEIGHT]

// IPV perpetration variable setup
    gen pipv_perp=0 if pv_perp_12mo!=.
    replace pipv_perp=1 if Q5_AR1_3==1 | Q5_AR2_3==1 | Q5_AR3_3==1
    label var pipv_perp "pipv perpetration"

    gen sipv_perp=0 if sv_perp_12mo!=.
    replace sipv_perp=1 if Q10_AR1_3==1 | Q10_AR2_3==1 | Q10_AR3_3==1 | Q10_AR4_3==1 | Q10_AR5_3==1 | Q10_AR6_3==1
    label var sipv_perp "sipv perpetration"

    gen ipv_perp=0 if sv_perp_12mo!=. | pv_perp_12mo!=.
    replace ipv_perp=1 if pipv_perp==1 | sipv_perp==1
    label var ipv_perp "ipv perpetration"

// Gender equality belief scale setup
    // 2-level items
    recode Q31A (1 2 = 1) (3 4 5 = 0) (98=.), gen(GEB_1)
    recode Q31B (1 2 = 1) (3 4 5 = 0) (98=.), gen(GEB_2)
```

```
    recode Q31C (1 2 = 1) (3 4 5 = 0) (98=.), gen(GEB_3)

    // reverse score such that higher = less equitable belief
    recode Q31A (1=5) (2=4) (3=3) (4=2) (5=1) (98=.), gen(q1)
    recode Q31B (1=5) (2=4) (3=3) (4=2) (5=1) (98=.), gen(q2)
    recode Q31C (1=5) (2=4) (3=3) (4=2) (5=1) (98=.), gen(q3)

    // drop from analysis those men missing
    drop if q1==. | q2==. | q3==.

    // create scale score
    alpha q1 q2 q3, gen(GEB_scale)
    corr q1 q2 q3

// Set up sociodemographic characteristics and other associated factors variables
    recode RACE_5 (1=1 "White")(4=2 "Hispanic") (2 3 5 = 3 "Black, Asian, Other/multiple race"), gen(race_recode)
    recode IDEO (-1 98=.) (1 2 = 1 "Liberal") (3 = 2 "Moderate") (4 5 = 3 "Conservative"), gen(ideo_recode)
    recode INCOME (1/10=1 "Less than 60k") (11/13=2 "60k-<100k") (14/18=3 "100k+"), gen(income_recode)
    recode MARITAL (1 6=1 "Yes") (2 3 4 5 = 0 "No") (98=.), gen (married)
    recode mental_cat (1 2 = 0 "normal/mild") (3 4 = 1 "mod/severe"), gen(mental2)
    recode Q36 (1 2 3 4 = 0 "No") (5 6 7 = 1 "Yes")(98=.), gen(drinking_6plus)
    recode Q37 (1 2 3 4 = 0 "No") (5 6 7 = 1 "Yes")(98=.), gen(druguse_6plus)
    recode Q11 (1 = 1 "Extremely safe") (2=2 "Quite safe")( 3 4 = 3 "Slightly/not at all safe") (98=.), gen(neighborhood_safe)
    recode Q22 (1 2 3 = 1 "Yes") (4 = 0 "No") (98=.), gen(own_gun)
        label var own_gun "Owns a gun (y/n)"
    // IPV victimization in past year
    replace ipv_12mo = IPV_PS_12mo if year==2023
    // Perpetration of any form of violence (against any victim) in past year
    gen perp_any_year=pv_perp_12mo
    replace perp_any_year=1 if sv_perp_12mo==1

// Assess for multicollinearity
    regress ipv_perp GEB_scale AGE i.race_recode i.ideo_recode i.EDUC_4 i.income_recode i.married i.year i.mental2 i.drinking_6plus i.druguse_6plus i.neighborhood_safe i.own_gun
        vif // remove education
    regress ipv_perp GEB_scale AGE i.race_recode i.ideo_recode i.income_recode i.married i.year i.mental2 i.drinking_6plus i.druguse_6plus i.neighborhood_safe i.own_gun
        vif // all VIFs <2 -- acceptable

// Numbers for text
    // Unweighted sample Ns
```

```
    di _N
    tab year

    // ATWA summary info
    svy: tab GEB_1
    svy: tab GEB_2
    svy: tab GEB_3
    svy: mean GEB_scale
    estat sd
    codebook GEB_scale

    // Test for variation over time
    svy: tab ipv_perp year, col ci
    svy: prop ipv_perp, over(year) coefl
    test _b[1.ipv_perp@2021bn.year]=_b[1.ipv_perp@2022.year]
    test _b[1.ipv_perp@2021bn.year]=_b[1.ipv_perp@2023.year]
    test _b[1.ipv_perp@2022.year]=_b[1.ipv_perp@2023.year]

    svy: mean GEB_scale, over(year) coefl
    test _b[c.GEB_scale@2021bn.year]=_b[c.GEB_scale@2022.year]
    test _b[c.GEB_scale@2021bn.year]=_b[c.GEB_scale@2023.year]
    test _b[c.GEB_scale@2022.year]=_b[c.GEB_scale@2023.year]

    // Share of all perpetrators who perpetrate against a partner
    svy: tab perp_any_year, ci obs
    svy: tab ipv_perp perp_any_year, col ci


// Table 1
    svy: tab ipv_perp, ci obs
    svy: tab pipv_perp, ci obs
    forvalues x=1/3 {
        replace Q5_AR`x'_3=0 if pipv_perp==0
        svy: tab Q5_AR`x'_3, format (%9.3f)
        }
    svy: tab sipv_perp, ci obs
    forvalues x=1/6 {
        replace Q10_AR`x'_3=0 if sipv_perp==0
        svy: tab Q10_AR`x'_3, format (%9.3f)
        }


// Table 2
    // Overall column
    svy: mean GEB_scale
        estat sd
    foreach v in AGE7 race_recode ideo_recode EDUC_4 income_recode married
 LGB_2 year {
        svy: tab `v', obs
        }
    foreach v in mental2 drinking_6plus druguse_6plus neighborhood_safe
own_gun  {
```

```
        svy: tab `v', obs
        }
    // Perpetration yes/no columns
    svy: mean GEB_scale, over(ipv_perp) coefl
        estat sd
        test  _b[c.GEB_scale@0bn.ipv_perp] = _b[c.GEB_scale@1.ipv_perp]
    foreach v in GEB_1 GEB_2 GEB_3  {
        svy: tab `v' ipv_perp, col obs
        }
    foreach v in AGE7 race_recode ideo_recode EDUC_4 income_recode married LGB_2 year {
        svy: tab `v' ipv_perp, col obs
        }
    foreach v in mental2 drinking_6plus druguse_6plus neighborhood_safe own_gun  {
        svy: tab `v' ipv_perp, col obs
        }


// Table 3
    svy: logit ipv_perp GEB_scale
    svy: logit ipv_perp GEB_scale AGE i.race_recode i.ideo_recode i.income_recode i.married i.LGB_2 i.year
    svy: logit ipv_perp GEB_scale AGE i.race_recode i.ideo_recode i.income_recode i.married i.LGB_2 i.year i.mental2 i.drinking_6plus i.druguse_6plus i.neighborhood_safe i.own_gun

    // Model with addition of past-year IPV victimization - only reported in text
    svy: logit ipv_perp GEB_scale AGE i.race_recode i.ideo_recode i.income_recode i.married i.LGB_2 i.year i.mental2 i.drinking_6plus i.druguse_6plus i.neighborhood_safe i.own_gun i.ipv_12mo, or


// Post-hoc analysis - sexual identity stratified results
    svy: tab ipv_perp LGB_2 , col format(%9.3f)
    svy: mean GEB_scale, over(LGB_2) coefl
    test _b[c.GEB_scale@1bn.LGB_2]= _b[c.GEB_scale@2.LGB_2]

    // Appendix Table 1
    svy, subpop(if LGB_2==2): logit ipv_perp GEB_scale, or
    svy, subpop(if LGB_2==1): logit ipv_perp GEB_scale, or
    svy, subpop(if LGB_2==2): logit ipv_perp GEB_scale AGE i.race_recode i.ideo_recode i.income_recode i.married i.year, or
    svy, subpop(if LGB_2==1): logit ipv_perp GEB_scale AGE i.race_recode i.ideo_recode i.income_recode i.married i.year, or


// Post-hoc analysis - separately run phyical & sexual IPV models
    svy: logit pipv_perp GEB_scale, or
    svy: logit sipv_perp GEB_scale, or

```
