# Supplementary material for: Are men’s gender equality beliefs associated with self-reported intimate partner violence perpetration? A state-level analysis of California men
Source: PLoS One. 2025 Jan 15;20(1):e0315293. doi: 10.1371/journal.pone.0315293 (PMC11734971; doi:10.1371/journal.pone.0315293)
Supplement: Attachment — Submitted filename: Referee report_2024_09_23.docx [file pone.0315293.s003.docx]

Referee report

This study uses survey data from California to test for an association between men’s own beliefs on gender equality and their self-reported IPV perpetration in the past year. In doing so, this study expands on prior work that describes society-level patriarchal values and IPV outcomes to instead consider how individual beliefs correspond to individual perpetration. The authors find that, among the 2% of their sample that self-reports perpetrating physical and sexual violence against an intimate partner in the past year, views supporting gender inequality are more prevalent. They further provide evidence that these correlate to policy-relevant characteristics such as gun ownership and substance abuse.

The paper is clearly written and conveys a cohesive and coherent argument. My comments below are intended to strengthen the manuscript as written rather than change anything the authors are doing. Major/substantive comments should improve the work’s clarity, avoid overstating conclusions, and address common reader questions and concerns. The paper should also be careful to remove any instances of causal language for this purely associative/correlative study.

Major/substantive comments

- Be sure to carefully distinguish IPV perpetration from IPV perpetration *self-reporting.* My first instinct upon seeing that 2% of the sample—while this does reflect a large number of individuals overall—is that this feels like an underestimate. Much literature discusses the willingness to self-report risky and harmful behaviors. How does the selection in who in your sample is willing to self-report perpetrating IPV impact your findings? I recommend both engaging with this literature, discussing what it means for your sample and generalizability, and tempering language when discussing perpetration (in favor of self-reported perpetration).
- Why do you limit IPV perpetration to physical and sexual violence? Does CalVEX include information about emotional, psychological, economic, spiritual, etc. abuse? These more poorly understood forms of violence may be less subject to self-reporting bias compared to IPV that corresponds more directly to illegal behavior (assault, sexual assault, etc.).
- I would appreciate more discussion of the choice of additional variables of interest. It’s unclear to me (though I could be easily convinced!) why perceptions of neighborhood safety might be associated with either gender equality beliefs or IPV perpetration.
- Line 364: you say that you analyze IPV perpetration association separately for non-heterosexual men in your sample and find differences but do not report results. While only 11% of the sample is non-heterosexual, this is still a substantial number that could be interpreted at least via descriptives, if not rigorously with statistical significance due to statistical power concerns. There is a strong argument to be made that, while queer/LGBTQ IPV is still connected to patriarchy (see Messinger, 2017), it is still fundamentally different when violence generated by patriarchal values is perpetrated by men against women versus by men against other men.
- The authors should demonstrate a lack of multicollinearity in the included demographic factors and additional variables of interest, perhaps via a correlation matrix.
- Please present p-values/confidence intervals backing up the claim that reductions in perpetration are decreasing year over year.
- Line 68-69: “Yet, we lack data in understanding if men’s views of women and gender equality are actually associated with IPV perpetration.” I think this claim should be tempered—we do know a lot about the role of patriarchy in generating patterns of DV and IPV, and this connection has been central to the movement fighting violence against women for decades. However, this is an aggregate and society-level connection. Perhaps it is more appropriate to say that we know less about how *individual* men’s views on gender equality correspond to individual perpetration of IPV in men’s own intimate relationships.
- On line 197 you state “no men declined to answer the IPV perpetration question,” but the subsequent table 1 includes a number of IPV metrics. How was this question structured if it is a single question but elicited this much detail on individual forms of IPV perpetration?
- Please remove all causal language from the manuscript (e.g., lines 315 and 336). Your methods are descriptive/correlative and should not be interpreted causally.

Minor comments

- Review the piece for grammar and spelling throughout.
- Be sure to spell out any acronyms at their first instance.
- Some sentences are far too long (e.g., line 56-61) to be easily parsed.
- Line 295-297 is unclear.
- Does CalVEX include data on violence perpetrated against former intimate/sexual partners?
- Why is the conclusion in a different vertical spacing format?
